# Community-Based Antiretroviral Therapy (ART) Delivery for Female Sex Workers in Tanzania: 6-Month ART Initiation and Adherence

**DOI:** 10.1007/s10461-019-02549-x

**Published:** 2019-06-13

**Authors:** W. Tun, L. Apicella, C. Casalini, D. Bikaru, G. Mbita, K. Jeremiah, N. Makyao, T. Koppenhaver, E. Mlanga, L. Vu

**Affiliations:** 1 HIV and AIDS Program, Population Council, Washington, DC USA; 2 HIV and AIDS Program, Population Council, Dar Es Salaam, Tanzania; 3 Jhpiego, Dar Es Salaam, Tanzania; 4 Jhpiego, Njombe, Tanzania; 5 National Institute for Medical Research, Mwanza Research Centre, Mwanza, Tanzania; 6 National AIDS Control Programme (NACP), Ministry of Health, Community Development, Gender, Elderly and Children, Dar Es Salaam, Tanzania; 7 United States Agency for International Development (USAID), Dar Es Salaam, Tanzania; 8 HIV and AIDS Program, Population Council, 4301 Connecticut Avenue, NW, Suite 280, Washington, DC 20008 USA

**Keywords:** Community-based ART, ART initiation, Adherence, Female sex workers (FSW), Tanzania

## Abstract

We conducted an implementation science study of a community-based ART distribution program for HIV-positive female sex workers (FSW) whereby clients received ART services through community-based mobile and home-based platforms. We compared 6-month treatment-related outcomes in the community-based ART arm (N = 256) to the standard facility-based ART delivery arm (N = 253). Those in the intervention arm were more likely to have initiated ART (100.0% vs. 71.5%; p = 0.04), be currently taking ART at the 6-month visit (100.0% vs. 95.0%; p < 0.01), and less likely to have stopped taking ART for more than 30 days continuously (0.9% vs. 5.7%; p = 0.008) or feel high levels of internalized stigma (26.6% vs. 39.9%; p = 0.001). In the adjusted regression model, internalized stigma (adjusted OR [aOR]: 0.5; 95% CI 0.28–0.83) and receiving community-based ART (aOR: 208.6; 95% CI 12.5–3479.0) were significantly associated with ART initiation. Community-based ART distribution model can improve linkage to and adherence to ART over standard facility-based ART programs for FSWs.

## Introduction

Linkage to HIV care and treatment, retention in care, and attainment of viral suppression is a challenge for people living with HIV in Tanzania, as in many other resource-limited settings [1–4]. Challenges to service access include health services-related barriers (e.g., poor quality of care, rigid clinic policies, stock-outs of supplies), providers’ stigmatizing attitudes and discrimination, psychosocial and emotional challenges (e.g., difficulty accepting HIV diagnosis, fear of being known as HIV positive, reluctance to access services while healthy), indirect costs (e.g., transportation costs, travel time to clinics), and perceptions and knowledge about antiretroviral (ARV) drugs (e.g., fear and myths about ARVs, toxicity of ARVs) [3–6]. The challenges are even greater for female sex workers (FSW), a highly stigmatized community [7–10].

FSWs in Tanzania are disproportionately affected by HIV, where the estimated HIV prevalence among FSWs is 26.6%, compared to a prevalence of 6.5% among general population of adult women [11, 12]. In Tanzania, with the increasing availability of antiretroviral therapy (ART) and the implementation of the national guidelines to treat all those diagnosed with HIV, the proportion of adult women living with HIV who are currently on ART is fairly high at 73% (57–87%) [13]. Furthermore, retention in ART services is also high, ranging from 83.7% among HIV positive females who initiated ART to 92.9% among HIV-positive females who know their HIV status [14, 15]. The lower estimate is based on routine service data and the higher estimate is based on self-reported use of ART. Research globally (including Africa) shows sub-optimal current ART usage among HIV-positive FSWs, ranging from 38 to 52% [7, 16, 17]. Data on ART uptake and treatment outcomes among FSWs in Tanzania is limited. A recent study among community-recruited FSWs in Tanzania indicated that 31% were previously aware of their HIV status, out of which 69% were on ART, and of those on ART, 70% were virally suppressed [18]. Evidence from studies with FSWs in Tanzania and other African countries have documented the challenges that FSWs face in accessing HIV care and treatment services, including difficulty reaching distant clinics, out-of-pocket costs associated with care, lack of knowledge and misperceptions of treatment, lack of respect for dignity and confidentiality, and persistent stigma associated with sex work and HIV [7–10].

Therefore, alternatives to mainstream health facility-based services are needed to ensure that FSWs are diagnosed early and access HIV treatment. One such approach is community-based service delivery interventions, which include strategies such as community outreach, mobile services, peer educators, community health workers, and community support groups. There is increasing evidence that community-based HIV testing and counseling (HTC) and ART distribution can improve HIV testing and treatment outcomes such as high rates of HTC uptake, high proportion of first-time testers, linkages to care, and ART initiation and retention [19–23]. In 2014, the World Health Organization recommended several solutions to improve access to ART for key populations, including decentralization of clinical services to sites located near hotspots, flexible clinic hours to accommodate those with non-traditional work hours, emergency drug pick-ups, client-held records to accommodate mobile clients, and non-judgmental staff attitudes [24].

To address barriers to access to HIV treatment among FSWs in Tanzania and in line with evidence from other countries, we conducted an implementation science study of a community-based ART distribution program for HIV-positive FSWs whereby HIV testing, care and ART services were provided by trained ART providers through community-based HIV testing and counseling (CBHTC) mobile and home-based platforms. This project is the first community-based ART service delivery strategy for FSWs using a CBHTC mobile and home-based platform in Tanzania. This CBHTC+ intervention is being implemented by Sauti project (a 5-year USAID-funded key and vulnerable population program led by Jhpiego). The research was conducted by the Population Council under the USAID-funded Project SOAR in collaboration with the National AIDS Control Programme (NACP); Ministry of Health, Community Development, Gender, Elderly and Children; and the National Institute of Medical Research (NIMR), Mwanza Research Centre. This paper reports on the comparison of ART initiation (primary outcome) and 6-month treatment-related outcomes (secondary outcomes) in the intervention and the standard ART delivery arms.

## Methods

### Study Design

A quasi-experimental prospective cohort study was conducted to examine differences in treatment outcomes between the intervention and comparison arms. The study entailed a baseline behavioral survey and a 6-month follow-up study visit (behavioral survey and viral load testing). A 12-month follow-up visit will be conducted 12 months after the baseline visit (this data is not yet collected at the writing of this paper).

### Study Sites

The study was conducted in Njombe (intervention) and Mbeya (comparison) regions. Njombe was purposely selected because of its high HIV burden (32.9% in Iringa, of which Njombe was part until 2012) among FSWs [12], high estimated number of FSWs (3,871) [25], and was a priority region for the donor. A region where Sauti was similarly operating its CBHTC+ mobile and home-based platforms and with a sufficiently high HIV prevalence and high number of FSWs was need to serve as the comparison site. The selected region, Mbeya, has an HIV prevalence of 29.2% [12], and an estimated large FSW population size (10,152) [25]. Both regions are part of the “Southern Highlands transportation corridor”, a major trucking route, and host many mobile seasonal workers, both of which make it ripe for a large sex work community. There are, however, some important differences. Mbeya region is four times larger than Njombe (approximately 2.7 million vs. 700,000) [26], has a higher proportion with no education among women (16.2% vs. 8.2%) [11], and is more urbanized (33% vs. 24%) [27]. Further, according to the 2013 bio-behavioral survey, FSWs in Mbeya compared to those in Iringa are slightly younger, and had higher risk-taking behaviors and vulnerabilities to HIV (e.g., higher inconsistent condom use and average number of paid partners per day, and higher proportions never HIV tested). Lastly, Mbeya region has lower ART care and treatment clinic (CTC) coverage compared to Njombe as of 2014 (2.29 vs. 3.79 CTC per 1000 infected adults) [2].

### Intervention Description

#### Intervention Arm (Njombe)

The intervention was developed in close consultation with in-country partners, including Sauti, NACP, USAID and the Njombe Regional Health Medical Team. The intervention aligned closely with the National ART guidelines on the management of HIV and the national community-based HIV and AIDS services (CBHS) guidelines (in draft at the time of intervention development). Using an implementation science approach [28], this intervention was developed through a participatory and iterative process involving the aforementioned stakeholders to identify gaps and develop strategies that would be be acceptable to the target population, and supported by the NACP thus helping to ensure research uptake and sustainability of the intervention if found to be effective. The intervention was built upon Sauti’s existing Community-based HIV Testing and Counseling Plus (CBHTC+) intervention, which includes the following services for FSWs: HTC, STI screening and periodic presumptive treatment, escorted referrals of HIV positive clients to HIV treatment facilities, condom promotion and provision, family planning counselling and methods, referrals for cases of gender-based violence, TB screening, and alcohol and drug screening. Providers directed presumptive TB clients and those using drugs or alcohol to referral services by using an onsite available directory of the surrounding facilities. These services were offered to all FSWs in both the intervention and the comparison arm sites. Table 1 outlines the services offered in the intervention and comparison arms, where the latter refers to the standard of care. For the intervention arm, the community-based ART delivery was added. Specifically, clinical staff were recruited to form the community-based health services team who provided the ART services through the CBHTC mobile and home-based platform. As required by the government, clients must register for ART at a CTC site and must have a CTC number. For the purpose of this study, all CBHTC “sites” were considered “satellite” facilities and linked to a government-managed CTC in order to obtain a CTC number and ensure ARV supply. The team was trained on recruitment of eligible candidates to the study, assessing client’s readiness for ART initiation, ART delivery, ART adherence counselling, and protocol for when clients should be referred to facility-based ART services (e.g., complications, opportunistic infections or pregnancy). Each team comprised one clinician and two nurses both trained in HTC and ART services, and at least three peer educators.

**Table 1:** Services provided in the intervention and comparison arms

Intervention arm (Njombe)	Comparison arm (Mbeya)
ART services provided at the community level by a CBHTC+ team (less stigmatizing services specific for key populations) as per national guidelines	ART services provided at government designated ART Care and Treatment Centres (CTC) by providers as per national guidelines for general population services
Enrolment in care through CBHTC+ sites	Enrolment in care at government CTC sites
Standard three sessions of adherence counselling at CBHTC+ sites	Standard three sessions of adherence counselling at government CTC sites
Drug pick-ups at client’s convenient and safe space from CBHTC+ teams	Drug pick-ups at government CTC sites
Drug supply: 1-month supply at first visit; 2-month supply at month 1 visit; 3-month supply at third visit and thereafter	Drug supply: typically 1 month supply; If deemed stable by clinician after first 6 months, a patient may be given drug refill appointment of 2–3 months interval
Providers trained on *test and start* as well as community-based ART delivery	Government standard practice regarding ART for key populations (*test and start*) as indicated in the guidelines
Service provided close to community; easy to access, less crowded, flexible time/days	Service provided at limited number of static CTC facilities. Longer commuting, and often more crowded, fixed working hours/days
Peer educators stayed in regular contact with participants through text messaging and *WhatsApp* (a smart phone messaging application) and met them on monthly basis to assess their adherence, side effects and provide peer support.	No follow-up contact in between CTC visits

Services provided in both arms	
If clients require, peer escort services will be available to access ART	
Standard client assessment and referrals to family planning and other services (TB screening and testing, STI testing and treatment, mental health support)	
Viral load and CD4 testing according to national guidelines	
Enhanced adherence counselling (three sessions) when treatment failure is suspected; this is conducted by a counsellor to help clients overcome factors contributing to treatment failure	

#### Comparison Arm

Enrolled participants were referred to government-designated ART service CTC facilities for current standard-of-care ART services, which entailed test and start per the national guidelines. Patients return for monthly visits for the first 6 months for evaluation and drug refills as well as adherence counseling. The main difference between the two arms is that the comparison arm did not provide any community-based ART services.

### Study Population and Sample Size

Eligible participants were females aged 18 years and above who sold sex for money or goods in the past 6 months, HIV positive and not currently on ART, and planning to reside in the region of recruitment for the next 12 months (or willing to return to recruitment district every 3 months for refill and check-up). Because community-based ART was delivered to stable clients, the World Health Organization (WHO) clinical stage was assessed at recruitment, and FSWs with stages 3 or 4 (with symptoms) were excluded and referred to government-designated ART facilities. Positive FSWs not on ART included: (i) newly diagnosed, (ii) previously diagnosed but not registered in care, (iii) in care but not on ART, and (iv) previously on ART but stopped ART for at least 3 months by the date of enrolment.

We estimated a sample size for the study to detect a difference of 20% points with 80% power in the key outcomes of ART initiation, ART retention, and viral suppression between the two arms at 12 months post-intervention at the p < 0.05 level and accounting for design effect of 1.5 and loss to follow-up (LTFU) of 20%. Based on Sauti’s routine programming data, we anticipated that 90% of the study participants in the intervention and 70% in the comparison arm would be initiated, and that 90% and 70% of those who initiated would be retained in care, and 80% and 60% of initiates would achieve viral suppression, respectively. The final sample size was determined as 300 per arm for the outcome of viral suppression, and 193 per arm for the outcome of initiation and retention.

### Procedure and Measurement

Study participants were actively recruited in July to October 2017 through: (i) community-based HTC in hotspots, (ii) contacting FSWs who were previously diagnosed by Sauti HTC services but not yet on treatment; (iii) brochures and announcements at targeted health facilities, peer support groups, and HTC sessions through the Sauti prevention team. Previously diagnosed persons were tested again to confirm their HIV status prior to starting ART.

The survey elicited information on demographics, HIV-related risk behaviors, HIV testing history, health status, sexual abuse, self- and external stigma, and enrollment into ART (for those who knew their HIV status for at least a month prior to the survey). Participants were asked if they could be contacted for a 6-month and 12-month follow-up visits. The follow-up visits entail the same behavioral survey with additional questions regarding ART uptake, adherence, and experience with HIV treatment services; the follow-up visits also included viral load testing. Interviews were conducted face-to-face in Swahili using handheld devices in private venues such as the participant’s or a peer educator’s home or rented room in a guesthouse. At each study visit, each participant received 10,000 Tanzanian shillings (about 4.5 USD) to offset the costs of travel and time and to reduce loss to study follow-up.

At the follow-up visits, participants were considered linked to care if they self-reported having registered in HIV care at a Care and Treatment Center (CTC) or Sauti CBHTC+ ART program. ART initiation was based on self-report of having been prescribed ARVs by a provider and started taking them. Adherence was measured in two different ways: whether they missed any ARV dose in the past 7 days and whether they stopped taking ART for more than 30 days continuously. Internalized HIV-related stigma was measured using a validated six-item scale, which assessed participants’ feelings of shame and guilt because of living with HIV (e.g., “I sometimes feel worthless because I am HIV positive”) [29]. Each item had two responses: “agree” and “disagree”. A composite index was computed and dichotomized at the median (low vs. high).

### Analysis

All analysis was performed using Stata analysis software (Version 14.1, College Station, Texas). Comparison of the baseline characteristics of the two arms, the comparison of the lost to follow-up participants to those retained in the study, and the comparison of treatment-related outcomes (e.g., ART initiation, currently on ART, missing dose in past 7 days, stopped taking ART for > 30 days continuously) in the two arms was analyzed using Chi square and Fisher’s exact tests for categorical variables and T-tests for continuous variables.

The main outcome for multivariate analysis was ART initiation. Multivariate analysis was performed to examine whether participation in the community-based ART intervention was associated with ART initiation at the 6-month follow-up. Because 100% of participants in the intervention arm started ART, this caused a complete separation in multiple logistic regression. Complete separation is defined as the outcome of each subject in the data set being perfectly predicted. We used an alternative to logistic regression called firth logistic. Firth logistic regression introduces a likelihood penalty that solves the separation problem [30, 31]. The selection of independent variables was initially determined through literature, theoretical concepts, and their levels of significance during bivariate analysis. We included key socio-demographic characteristics (age, education, marital status, and mobility) as covariates. As stigma is a critical barrier to accessing care, we also explored how it affects treatment initiation. Bivariate analysis was conducted to examine factors associated with ART initiation at the 6-month follow-up. Variables significant at p ≤ 0.15 in bivariate analysis were included in the final multivariate analysis [32, 33]. Unadjusted and adjusted odds ratios and 95% confidence intervals are reported.

### Ethical Considerations

The study was approved by the Population Council Institutional Review Board (USA), the National Institute for Medical Research, Medical Research Coordinating Committee (Tanzania), and the Mbeya Consultant Hospital, Mbeya Medical Research and Ethics Review Committee (Tanzania).

## Results

### Recruitment and Participant Characteristics

A total of 342 (intervention) and 332 (comparison) were screened for the study, and 33 and 24 were found ineligible, respectively. Reasons for ineligibility included not selling sex in past 6 months, being younger than 18, being pregnant, and currently taking ART or stopped < 3 months ago. Of those found to be eligible, all agreed to participate. Table 2 describes baseline characteristics of the intervention (N = 309) and comparison (N = 308) arms. Participants in the intervention arm were significantly younger (29 years vs. 32 years), had completed more years of education (20.4% vs. 7.4% completed secondary education or more), more likely to have 51–100% of their income from sex work (38.5% vs. 23.7%), have more than one non-paying partner in the past month (32.4% vs. 11.7%), have travelled out of the region to sell sex in the last 6 months (35.6% vs. 10.4%), and been sexually abused in the last 6 months (38.2% vs. 5.0%). The two groups were similar with regard to marital status, and number of living children.

**Table 2:** Characteristics of the study population in the intervention (N = 309) and comparison (N = 308) arms at baseline

Variable	Intervention (n = 309) n (%)	Comparison (n= 308) n (%)	Total (N = 617) n (%)
Age^***^			
Median (IQR)	29 (25–35)	32 (26–39)	30 (25–37)
Education^***^			
None	23 (7.4)	42 (13.6)	65 (10.5)
Some primary	223 (72.2)	244 (79.2)	467 (75.7)
Secondary or more	63 (20.4)	22 (7.2)	85 (13.8)
Marital status			
Never married	185 (59.9)	163 (52.9)	348 (56.4)
Married, living with a husband	22 (7.1)	35 (11.4)	57 (9.2)
Divorced/widowed/separated	102 (33.0)	110 (35.7)	212 (34.4)
Number of living children			
None	55 (17.8)	51 (16.6)	106 (17.2)
One	115 (37.2)	75 (24.4)	190(30.8)
More than one	139 (45.0)	182 (59.0)	321 (52.0)
Percentage of income from sex work (past 6 months)^***^			
Under 25%	80 (25.9)	72 (23.4)	152 (24.6)
26–50%	110 (35.6)	163 (52.9)	273 (44.3)
51–100%	119 (38.5)	73 (23.7)	192 (31.1)
Number of non-paying sex partners (past 1 month)***			
None	89 (28.8)	173 (56.2)	262 (42.5)
One	120 (38.8)	99 (32.1)	219 (35.5)
More than one	100 (32.4)	36 (11.7)	136 (22.0)
Travelled out of the region to sell sex (past 6 months)^***^			
No	199 (64.4)	276 (89.6)	475 (77.0)
Yes	110 (35.6)	32 (10.4)	142 (23.0)
Being sexually abused (at least once, past 6 months)***			
Yes	118 (38.2)	5 (1.6)	123 (19.9)
No	191 (61.8)	303 (98.4)	494 (80.1)
Time since HIV diagnosis^***^			
Newly diagnosed today	56 (18.1)	138 (44.8)	194 (31.5)
Diagnosed within a month	214 (69.3)	112 (36.4)	326 (52.8)
Diagnosed more than a month ago	39 (12.6)	58 (18.8)	97 (15.7)

*IQR* Interquartile range

***p ≤ 0.001

### Loss to Follow-Up

Of the 309 (intervention) and 308 (comparison) baseline participants, a total of 256 (82.8%) and 253 (82.1%) completed the 6-month follow-up visit, respectively. Among the 53 LTFU participants in the intervention arm, three participants died (confirmed by Sauti CBHTC+ clinical team), five refused to continue in the study, 42 were not reachable, and three transferred out to another CTC. Among the 55 LTFU in the comparison arm, one died (confirmed by NIMR Regional Research Coordinator), three refused to continue in the study, 37 were not reachable, and 14 were wrong telephone numbers. Comparison of those who remained in the study and the 104 participants LTFU indicated that there were no significant differences with regard to age, marital status, education, number of living children, income from sex work, average monthly income, and traveling outside the region to sell sex. (Data not shown) When stratified by study arm, there were no significant differences between the LTFU and those who remained in the intervention arm. (Table 3) However, in the comparison arm, LTFU were slightly younger and less likely to have travelled outside of the region for sex work in the past 6 months.

**Table 3:** Comparison of participants lost to follow-up (LTFU) and participants remaining in the study at the 6-month follow-up visit by study arm

	Intervention			Comparison		
	LTFU (n = 53)	Remained (n = 256)	*p* value	LTFU (n = 55)	Remained (n = 253)	p value
Age						
Median (IQR)	30 (23–35)	28 (25–35)	0.52	30 (25–35)	33 (27–39)	0.045
Education						
None	3 (5.7)	20 (7.8)	0.81	11 (20.0)	31 (12.3)	0.31
Some primary	38 (71.7)	185 (72.3)		40 (72.7)	204 (80.6)	
Secondary or more	12 (22.6)	51 (19.9)		4 (7.3)	18 (7.1)	
Marital status						
Never married	28 (52.8)	157 (61.3)	0.16	34 (61.8)	129 (51.0)	0.35
Married/living with a husband	2 (3.8)	20 (7.8)		5 (9.1)	30 (11.9)	
Divorced/widowed/separated	23 (43.4)	79 (30.9)		16 (29.1)	94 (37.1)	
Number of living children						
None	11 (20.8)	44 (17.2)	0.66	12 (21.8)	39 (15.4)	0.50
One	17 (32.1)	98 (38.3)		12 (21.8)	63 (24.9)	
More than one	25 (47.2)	114 (44.5)		31 (53.4)	151 (59.7)	
Percentage of income from sex work (past 6 months)						
Under 25%	15 (28.3)	65 (25.4)	0.82	14 (25.5)	58 (22.9)	0.92
26–50%	17 (32.1)	93 (36.3)		28 (50.9)	135 (53.4)	
51–100%	21 (39.6)	98 (38.3)		13 (50.9)	60 (23.7)	
Average monthly income						
Median (IQR)	100000 (60–200000)	120000 (50–300000)	0.33	80000 (50–150000)	90000 (40–150000)	0.91
Travelled out of the region to sell sex (past 6 months)						
No	36 (67.9)	163 (63.7)	0.35	45 (81.8)	231 (91.3)	0.037
Yes	17 (32.1)	93 (36.3)		10 (18.2)	22 (8.7)	

### Treatment-Related Indicators

Table 4 describes the 6-month treatment outcomes of the participants in the intervention and comparison arms. Those in the intervention arm were more likely to have registered in HIV care (100.0% vs. 72.7%; p < 0.001), initiated ART (100.0% vs. 71.5%; p = 0.04), be currently taking ART at the 6-month visit (100.0% vs. 95.0%; p < 0.001), and less likely to have stopped taking ART for more than 30 days continuously (0.9% vs. 5.7%; p = 0.008). Additionally, those in the intervention arm were less likely to feel high levels of internalized stigma compared to those in the comparison arm (26.6% vs. 39.9%; p = 0.001).

**Table 4:** Comparison of key outcomes between intervention (n = 256) and comparison (n = 253) arms at the 6-month follow-up visit

Variable	Intervention arm % (n)	Comparison arm % (n)	p-value
Registration in HIV care/linkage to care	(n = 256)	(n = 253)	
Yes	100.0 (256)	72.7 (184)	<0.001
No	0 (0)	27.3 (69)	
ART initiation	(n = 256)	(n = 253)	
Yes	100.0 (256)	71.5 (181)	0.04
No	0 (0)	28.5 (72)	
Currently on ART (among those who initiated ART)^a^	(n = 254)	(n = 180)	
Yes	100.0 (254)	95.0 (171)	<0.001
No	0 (0)	34.2 (7)	
Missing at least a dose in past 7 days^a^	(n = 214)	(n = 152)	
Yes	17.3 (37)	16.4 (25)	0.83
No	82.7 (177)	83.6 (127)	
Stopped taking ART for more than 30 days continuously	(n = 214)	(n = 159)	
Yes	0.9 (2)	5.7 (9)	0.008
No	97.1 (212)	94.3 (150)	
Internalized HIV-related stigma	(n = 256)	(n = 253)	
Low	73.4 (188)	60.1 (152)	0.001
High	26.6 (68)	39.9 (101)	

^a^The smaller sample size is due to an error in a skip pattern which meant that some participants were not able to respond to this question

### Factors Associated with Treatment Initiation

The factors associated with ART initiation are shown in Table 5. In unadjusted analysis, we found that FSWs were significantly more likely to have initiated ART if they travelled out of the region to sell sex in the last 6 months, felt less internalized stigma, were diagnosed within a month ago (vs. the day of baseline), and received community-based ART. In multivariable analysis, only internalized stigma (adjusted OR [aOR]: 0.5; 95% CI 0.28–0.83) and receiving ART from a community-based service (aOR: 208.6; 95% CI 12.5–3479.0) remained significantly associated.

**Table 5:** Factors associated with and effects of the intervention on ART initiation at 6 months (N = 509)

	ART initiation	
	OR (95% CI)	aOR (95% CI)
Age		
≤ 30	1.0	
> 30	0.7 (0.43–1.20)	N//A
Education		
None	1.0	
Some primary	1.0 (0.45–2.10)	N/A
Secondary or more	1.1 (0.63–1.93)	
Marital status		
Never married	1.0	
Married, living with a husband	1.3 (0.58–2.93)	N/A
Divorced/widowed/separated	1.1 (0.63–1.93)	
Travelled out of the region to sell sex (past 6 months)		
No	1.0	1.0
Yes	2.2 (1.01–4.73)^*^	0.6 (0.2–1.4)
Internalized HIV-related stigma		
Low	1.0	1.0
High	0.4 (0.23–0.63)^***^	0.5 (0.28–0.83)^**^
Time since HIV diagnosis		
Newly diagnosed today	1.0	1.0
Diagnosed within a month	2.7 (1.6–4.8)^***^	1.2 (0.6–2.1)
Diagnosed more than a month ago	1.8 (0.86–3.6)	1.4 (0.7–3.0)
Received community-based ART intervention		
No (Mbeya)	1.0	1.0
Yes (Njombe)	2.5 (1.9–3.3)	208.6 (12.5–3479.0)^***^

*p ≤ 0.05; **p ≤ 0.01; ***p ≤ 0.001

## Discussion

This is the first study to report on the treatment outcomes after community-based distribution of ART services to FSWs in Tanzania. Findings from this study demonstrate that ART provision through community-based distribution can lead to higher ART initiation rates with continued ART use and better adherence after 6 months, compared to standard facility-based ART provision.

We found linkage to care (i.e., registration in HIV care) to be 100% in the intervention arm, which was much higher than the comparison arm (72%). We also found that once they are registered in care, a large majority initiates ART, even among the comparison participants. In fact, in the intervention arm, all who were registered did indeed initiate ART. This is likely due to the innovative CBHTC+ team service model, which by design was actively involved in the recruitment, testing/retesting (if necessary), enrollment and ART initiation of the cohort participants by peer educators who are familiar with the cohort participants and their lifestyles.

Regression analysis supported these findings, showing that treatment initiation was independently associated with receiving community-based ART. This is not surprising, given that clients in the intervention arm received immediate adherence counseling and a 1-month ARV supply, unlike those in the comparison arm who received passive referral to treatment at government-designated ART centers, though offered to be escorted by Sauti peer educators. Lower internalized HIV stigma was also found to be independently associated with ART initiation. Studies have shown that internalized stigma can manifest in a variety of ways, including low self-esteem, self-isolation, shame, withdrawal from seeking health services, and depression [34–36], which in turn can impact HIV treatment-seeking behaviors [37]. This suggests that ART services need to adopt strategies to develop a healthy self-perception in relation to their HIV diagnosis and cope psychologically. Such strategies include linkage to peer educators/supporters who understand their lifestyle, peer support groups, educating oneself/others about HIV, viewing HIV as a manageable condition, and controlled and pre-emptive HIV disclosure [38–41].

Although both arms showed high proportions being currently on ART at 6 months (retention), the intervention arm had a significantly higher proportion. Additionally, almost no one in the intervention arm stopped taking ART for more than 30 days continuously since they initiated, unlike in the comparison arm where 5.7% stopped for a period. This is likely due to the active nature of the community-based ART intervention, which used peer educators to schedule appointments for ARV refills and monthly meetings to support adherence. These early results demonstrate that for a high-risk population that is less likely to access facility-based ART and more likely to be mobile, an active and flexible ART delivery system can attain remarkably high treatment outcomes. A qualitative study conducted among HIV-positive clients who were part of a community ART group, whereby members take turns collecting ARVs for group members found that reduced frequency of clinic visits and associated transportation costs were important in facilitating ART access [42]. Indeed, our community-based ART intervention reduces clinic visits and transportation costs, given that the ART team went to where the clients were for the regular clinical evaluations and ARV delivery. Another explanation for the higher rate of current ART usage may be the reduced stigma and discrimination they face when accessing ART services in the intervention arm, as they are not subject to stigma they may face when visiting CTCs. In fact, those in the intervention arm reported feeling less internalized stigma. This is not surprising since providers and peer educators in the intervention arm were specifically trained to provide sensitive, private, and confidential services to key and vulnerable populations. Additionally, the community-based services offer other services to the HIV negative FSWs so as to avoid the perception that Sauti clinical team is only providing ART services, thereby disclosing FSW HIV status.

The current ART usage percentage at 6 months among those who initiated ART in both arms is better than the percentage among HIV-positive women who initiated ART (83.7%) based on routine service data from 2015 in Tanzania as reported by Mee et al. [15]. The retention rate in our study may be slightly higher than the rate reported by Mee et al. for several reasons: i) our findings are 6 months since ART intiation whereas the Mee et al. study is 12 months since initiation; ii) Mee et al. study is based on routine service data, which often have missing data; and iii) ART services may likely have improved in the past four years since the period of the data from the Mee et al. study. The retention rate found in this study is also similar to rates found in other countries, particularly those supported by LINKAGES, a USAID-funded project implemented by FHI360, where there have been active referrals and support for FSWs to access ART, for example through community-based outreach, ART provision at drop-in centers, and support from peer educators and case managers [43]. The slightly but significantly lower current ART usage percentage in the comparison arm compared to the intervention arm in this study  may reflect some of the structural barriers faced by FSWs in accessing facility-based ART services, as previously reported [7–9]. Additionally, given the fairly low CTC coverage rate (i.e., number of CTC facilities per 1000 infected persons), it is not surprising the comparison arm shows lower ART usage percentage. Although the comparison arm had a lower percentage of current ART usage, it is still higher than what has been reported in other countries, including African countries among FSWs where the current ART usage ranges from 38 to 52% [7, 16, 17]. It is possible these rates in other countries have improved in the past few years since the publication of the findings, given the WHO *Test and Start* recommendation) and the increased provision of variations of community-based ART. The Government of Tanzania adopted this WHO recommendation in October 2017 of treating all persons upon HIV diagnosis.

In light of these positive findings around community-based ART, further discussions are needed around the current national policy requiring PLHIV to complete 6 months of facility-based ART and reach viral suppression prior to being transferred to community-based ART. This study shows that FSWs not currently on ART at the time of enrollment, can indeed be initiated on ART and continue to do so for 6 months. The “stability” requirement to be eligible for community-based ART may hinder the achievement of the second and third 95–95–95 goals as many key population members will have a hard time reaching “stability” due to facility-based related barriers they face. This study has several limitations. First, treatment outcomes were self-reported, and thus participants may have responded in the favorable direction due to social desirability. Second, the two regions were not randomly selected, which may reduce the comparability of the two arms. Although the analysis controlled for differences in certain factors such as demographics and other factors that might affect the outcome (time since diagnosis and internalized stigma), there were substantial differences in participant characteristics across sites and the analysis cannot control for other unknown factors. However, although the two regions differed on several factors (e.g., risk behaviors, gender-based violence), these variables were not associated with the outcome of interest (data not shown), and hence our analysis did not adjust for these other differences. Although the two regions were not selected randomly, the study’s strength lies in the fact that the intervention was implemented in the ‘real-world’ setting as opposed to a strict controlled setting in which generalizability would be reduced. The intervention was implemented how it would typically be implemented outside of the study setting, thus increasing the generalizability of the findings and the likelihood that the approach can be more readily incorporated to inform scale-up [28].

## Conclusions

As countries aim to reach the 95–95–95 targets, national HIV responses must seek to expand their strategies to effectively provide ART services. This study showed that a community-based ART distribution model can improve linkage to and adherence to ART over the standard facility-based ART program. Addressing internalized stigma should be an integral component of ART programs for FSWs. Further research is needed to understand the cost and cost-effectiveness of the community-based approach to assess its sustainability particularly in an environment with limited resources as well long-term adherence and retention of HIV clients enrolled in community ART.
